# Moringin, A Stable Isothiocyanate from *Moringa oleifera*, Activates the Somatosensory and Pain Receptor TRPA1 Channel In Vitro

**DOI:** 10.3390/molecules25040976

**Published:** 2020-02-22

**Authors:** Gigliola Borgonovo, Luciano De Petrocellis, Aniello Schiano Moriello, Simona Bertoli, Alessandro Leone, Alberto Battezzati, Stefania Mazzini, Angela Bassoli

**Affiliations:** 1 Department of Food, Environment and Nutrition-DeFENS, University of Milan, Via Celoria 2, I-20133 Milano, Italy; gigliola.borgonovo@unimi.it (G.B.); simona.bertoli@unimi.it (S.B.); alessandro.leone1@unimi.it (A.L.); alberto.battezzati@unimi.it (A.B.); stefania.mazzini@unimi.it (S.M.); 2 Endocannabinoid Research Group-Institute of Biomolecular Chemistry-CNR, Pozzuoli, I-87078 Napoli, Italy; luciano.depetrocellis@icb.cnr.it (L.D.P.); aniello.schianomoriello@icb.cnr.it (A.S.M.); 3 Epitech Group SpA, Saccolongo, 35030 Padova, Italy

**Keywords:** *Moringa oleifera*, moringin, TRPA1 ion channel, isothiocyanates

## Abstract

*Moringa oleifera* Lam. is a tropical plant widely used in traditional medicines and as a food supplement. It is characterized by the presence of glucosinolates and isothiocyanates; the stable isothiocyanate 4-[(α-l-rhamnosyloxy)benzyl]isothiocyanate (moringin) has been widely studied for its bioactivity as hypoglycemic, antimicrobial, anticancer and in particular for its involvement in nociception and neurogenic pain. Moringa extracts and pure moringin were submitted to in vitro assays with the somatosensory TRPA1 ion channel, proving that moringin is a potent and effective agonist of this receptor involved in nociceptive function and pain states. Moringin do not activate or activates very weakly the vanilloids somatosensory channels TRPV1,2,3 and 4, and the melastatin cooling receptor TRPM8. The comparison of moringin’s activity with other known agonists of natural origin is also discussed.

## 1. Introduction

### 1.1. Moringa oleifera Lam., A Multi-Purpose Tree

*Moringa oleifera* Lam., (family Moringaceae, order of Capparales), is a tropical and subtropical plant diffused worldwide. It has been used in traditional medicines since long time.

The plant is known with the common names of moringa, drumstick tree (from the long, slender, triangular seed-pods) or horseradish tree (from the pungent taste of the roots, resembling horseradish).

Some recent pharmacological studies appear to validate the claimed medicinal uses of the plant, primarily the leaves, which include antioxidant, anti-carcinogenic, anti-diabetic, anti-inflammatory and anti-hypertensive properties, as well as the ability to protect against hepatic damage. The phytochemistry and pharmacology of moringa has been recently reviewed [1,2].

Due to its nutrient constitution, *M. oleifera* is used in several regions to treat malnutrition. Its bitter and pungent taste sometimes limits the consumers’ acceptance level, especially in Western cultures not familiar with it. Recently some of us have studied the taste acceptability of moringa leaf power in meals, and it proved to be low, while an important hypoglycemic effect was well demonstrated by trials and evaluating the long-term effects on glycaemia. [3].

The characterization of *M. oleifera* leaf extract have recorded a total of 59 bioactive compounds, including amino and organic acids, nucleosides, glucosinolates, lignans, phenolic acids, and flavonoids [4].

Glucosinolates (GLSs) are secondary metabolites especially abundant in plants of the order of Capparales. Crucifer vegetables in addition to GLSs contain myrosinase, a specific thioglucosidase, located in separate cell compartments. When the enzyme and the substrates come in contact in an aqueous environment, for example due to tissue damage or chewing, myrosinase causes immediate hydrolysis of GLSs which produces sugars and an unstable aglycon fragments [5] that after structural rearrangements forms various compounds including isothiocyanates (ITCs). Both GLSs and ITCs are largely investigated for their medicinal effects.

**Figure 1:**
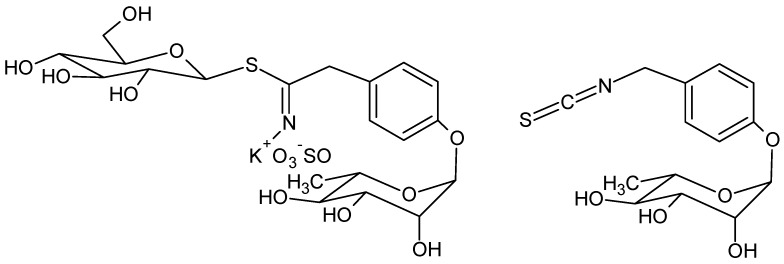
Structure of glucomoringin (left) and moringin (right).

Glucomoringin is the main GLS in moringa [6] and moringin is the corresponding ITC (Figure 1).

Due to the presence of the polar O-glycosylated phenol substituent, moringin is a solid, odorless and relatively stable compound at room temperature; these are uncommon features for most isothiocyanates obtained by crucifer plants, such as benzyl- and allylisothiocyanate from mustard which are usually small and volatile compounds, with a pungent smell. [7].

The biological activity of moringin extracts and moringin itself attracted the attention of a number of researchers in different areas. This phyto-compound possesses in fact a wide range of biological activities, including antioxidant [8], antimicrobial [9], antibacterial [6], antifungal and antiviral [10,11,12], and deterrent effects towards some insects. [13].

Moringin is also studied for medicine applications: beside the hypoglycaemic effects described above, it shows anti-inflammatory [14] and antitumor effect [15,16]. Moringin has shown potential in the stem cell-based therapeutic treatments in neurodegenerative diseases such as Parkinsons’ and Altzheimer’s diseases and it protects against neurodegenerative disorders [17,18].

A comparison between moringin and cannabidiol in the treatment of stem cells as a therapeutic approach for inflammation in neurodegenerative diseases has been also recently discussed [19]. Moringin has been shown to defends cerebral tissue and prevent severe damage induced by focal ischemia/reperfusion [20]; another study on the Amyotrophic Lateral Sclerosis (ALS) transgenic model revealed a significative interference of moringin with the pathophysiology mechanisms that induced the development of the disease. The moringin administration was able to delay the disease onset of about two weeks [21].

Recently the role of H_2_S release from glucosinolates/isothiocyanates as a potent mechanism of protective action in the cardiovascular compartment and in the nervous system has been reported [22], as well as the role of H_2_S-mediated pain-relieving effects of natural and synthetic isothiocyanates [23].

The involvement of moringin in nociception has been often reported. Potential analgesic activities, in vivo anti-arthritic and evidences of broad spectrum of anti-nociceptor effects were described in the literature. [24,25].

Recently Giacoppo and coworkers [26] reported the efficacy of 2% moringin cream treatment in reducing clinical and histological disease score, as well as in alleviating neuropathic pain. A formulation with moringin encapsulated in α-cyclodextrin showed anti-inflammatory effects in LPS activated macrophages, suggesting a therapeutic approach for inflammatory diseases. [27].

### 1.2. TRPA1 Channel in Nociception

Previously called ANKTM1 (ankyrin repeat-containing ion channel 1), Transient Receptor Potential type Ankyrin (TRPA1) channel is the only member of the TRPA sub-family reported in mammals [28]. TRPA1 is a member of the large TRP family of ion channels, is a Ca^2+^ permeable non-selective cation channel functioning in many different cell processes as polymodal sensor involved in the perception of several stimuli among which cold, pungent compounds, airborne irritants and cannabinoids as well as in neurogenic inflammatory responses and pain perception [29,30]. TRPA1 is expressed in neuronal dorsal root and trigeminal ganglion neurons [31] and non-neuronal tissues and cells such as skin keratinocytes [32,33] and airway epithelial cells [34]; its activation on trigeminal nerves may produce head pain [35].

TRPA1 is activated by a large number of electrophilic, pungent compounds such as isothiocyanates and unsaturated aldehydes [36], pungent compounds present in garlic [37,38], ketones and thiosulfinates common in many plants, particularly in *Brassicaceae* and are often characterized a by a bitter and pungent taste, and the plant-derived cannabinoid receptor agonists THC [39]. These compounds have very different structures, most of them may covalently modify intracellular cysteines and/or lysines opening the channel [40,41]. However, it has been reported the presence of alternative activation mechanisms so that TRPA1 may be activated by other plant compounds that not interact with cysteines [42]. Many of them, included *Moringa oleifera*, are important crops for human and animal diets and are also commonly used as traditional remedies. Allylisothiocyanate (mustard oil) is the reference agonist for TRPA1; other isothiocyanates as benzyl-, phenylethyl-, isopropyl-, and methyl-isothiocyanates have been proved to activate TRPA1 in vitro [43,44]. The role of TRPA1 channel in pain has been extensively reviewed [45,46].

### 1.3. Aim of This Work

The aim of this work is to assess the activity of moringin on TRPA1 ion channel. At this aim, we prepared extracts from leaves of *Moring oleifera* using solvents of different polarities. Moreover we isolated moringin (4-[(α-l-rhamnosyloxy)benzyl]isothiocyanate) from moringa leaves; extracts and isolated moringin were submitted to in vitro assays with cloned TRPA1 receptor. Moringin turned out to be a potent agonist of TRPA1. Other ion channels, as the vanilloids TRPV1-4 and TRPM8 were also tested giving very low (for TRPV1) or no measurable response.

## 2. Results

### 2.1. Preparation of Extracts and Isolation of Moringin

Leaves of *Moringa oleifera* were used as dry powdered leaves or frozen leaves. Starting from moringa powder we obtained four extracts using solvents with increasing polarity: hexane (E1), diethylether (E2), dichloromethane (E3), and ethanol (E4). For extracts E5 and E6, the extraction of moringa leaves was performed in order to optimize in situ the hydrolysis of glucomoringin in moringin by myrosinase. At this aim, frozen leaves were finely crushed at room temperature to free the enzyme before extraction with water and methanol respectively.

Similarly, moringin (purity >94%, HPLC) was obtained from the methanolic extract of frozen moringa leaves following a literature method [47]. The structure was confirmed by NMR analysis in comparison with literature data [13,48].

### 2.2. In Vitro Assays

Extracts (E1-6) and pure moringin were submitted to in vitro assays with the TRPA1 receptor.

Allylisothiocyanate (AITC) is conventionally used as the reference agonist for TRPA1 and was used to establish the relative efficacy for each tested compound. Antagonist/desensitizing behavior was also measured. The results are shown in Table 1.

Among the extracts from dry powder, only E1 and E2 show some activity whereas E3, E4 and E5 are inactive. The most active extract is E6, obtained from frozen leaves, with an efficacy of 99% and a potency of 1.3 µg/mL.

**Figure 2:**
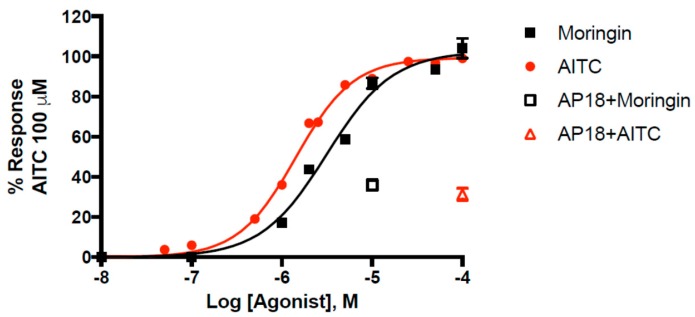
Dose-related effect of moringin and allylisothiocyanate (AITC) on elevation of intracellular Ca^2+^ in human embryonic kidney (HEK)-293 cells stably transfected with rat TRPA1 (rTRPA1-HEK-293). AP18 = 4-(4-chlorophenyl)-3-methyl-3-buten-2-one oxime. Data are expressed as percentages of the effect observed with AITC 100 µM.

Pure moringin has an efficacy of 102.6 ± 1.1% and a potency of 3.14 ± 0.16 μM that is comparable to that of AITC (EC_50_ = 1.43 ± 0.04 μM), in good agreement with what we previously reported [49]. Moringin has emerged as one of the most potent natural agonists of TRPA1 in vitro, with a potency very close to that of curcumin (EC_50_ = 3.3 μM) [50]. The selective TRPA1 channel antagonist AP18 (4-(4-chlorophenyl)-3-methyl-3-buten-2-one oxime) inhibited moringin and AITC-induced effects in TRPA1-HEK293 cells. We found that AP18 (at 50 μM), reduced elevation of intracellular Ca^2+^ in human embryonic kidney (HEK)-293 cells stably transfected with rat TRPA1 (rTRPA1-HEK-293) by 68.6 ± 3.0% and 64.1 ± 2.7% respectively by AITC (100 μM) and moringin (10 μM). The dose-response curve for moringin is shown in Figure 2.

**Figure 3:**
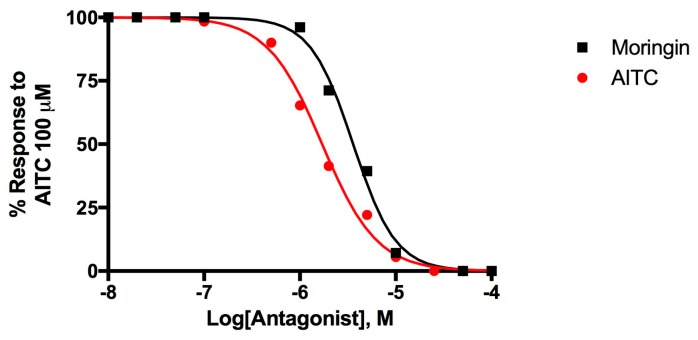
Dose-related effect of 5-min pre-incubation of rTRPA1-HEK-293 cells with moringin and allylisothiocyanate (AITC) on the AITC (100 µM)-induced elevation of intracellular Ca^2+^. Data are expressed as percentages of the effect observed with AITC (100 µM) alone.

Figure 3 shows the dose-response curves to evaluate the desensitizing behavior of moringin, a well-known effect for TRPA1 agonists [51,52].

Moringin **2** desensitizes TRPA1 with an IC_50_ = 3.60 ± 0.05 μM while AITC activates and subsequently desensitize TRPA1 with an IC_50_ = 1.71 ± 0.05 μM. Moringin has a very strong desensitizing effect which is typical of TRP channels, the effect of AITC 100 μM administered after a pre-incubation of TRPA1-HEK293 cells with moringin 50 μM, is completely inhibited.

Moringin is likely the active principle responsible of the activity of the alcoholic extract E6. Moringin is in fact detectable in this extract by TLC analysis and comparison with purified sample. The activity is much lower in E4, prepared from dried powder, where myrosinase has been inactivated and the hydrolysis of glucosinolate to give moringin is therefore very slow.

Moringin was then assayed with other receptors of the vanilloid family, i.e., TRPV1,2,3,4, and with the melastatin cooling receptor TRPM8. For TRPV1 the potency resulted weak and for the others the efficacy is lower than 10%. Results are shown in Table 2.

## 3. Discussion

The use of moringa and its extracts for medicinal purposes is well established; in particular, recent evidences for its analgesic activity has been reported. Pharmacological evaluation of non-polar and/or polar extracts was explored through several experimental nociception models as formalin test, carrageenan-induced paw edema and arthritis with subcutaneous injection of collagen in rats [24]. The authors found that both polar and non-polar extracts produced significant inhibition of nociceptive behavior.

Phytochemical analysis allowed to identify several molecules able to exert an analgesic effect, as kaempferol-3-glucoside, in the polar extract and fatty acids like chlorogenic and palmitic acid, among others, in the non-polar extract. In moringa polar extracts the antinociceptive activity was suggested to be modulated via opioid receptors at the central, but not peripheral, antinociceptive level [53].

Moringa leaves have also been reported to contain various types of flavonol glycosides, as well as kaempferol, rutin, and quercetin [54]. Additionally, these flavonols are TRPA1 agonists [55].

In this paper we report that moringin, the main stable isothiocyanate in moringa, is itself a very effective and potent agonist of TRPA1. This data reinforces the hypothesis that this ion channel could be one of the mediators of the analgesic effect of the plant. The activity of moringin is selective, since no other receptors of the TRPV vanilloid family nor the TRPM8 are activated.

**Figure 4:**
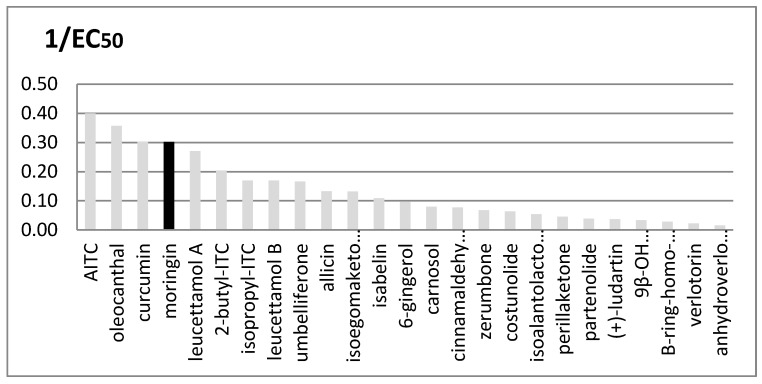
Comparison between in vitro rTRPA1′s activity of many natural known agonists. Potency is expressed as 1/EC_50_; EC_50_ values are given in μM units. Moringin from *Moringa oleifera* is shown in black.

Many other plants are rich in TRPA1 agonists, and their importance as functional ingredients against inflammation and pain is rapidly growing, particularly when the plant is a food plant and therefore a cheap and accessible source of such medicinal principles. We compared the EC_50_ values of several natural agonists reported for rTRPA1 (Figure 4).

It is interesting to note that the three most active compounds in this group, i.e., oleocanthal, curcumin and moringin, come from widely distributed food plants.

*Moringa oleifera* is a very promising source of functional bioactives for its large diffusion in tropical and sub-tropical areas and its ability to grow in arid climatic conditions where nutrition and health issues are both critical for populations.

Our data on extracts E1 and E2, obtained by hexane and diethylether, show that beside moringin, other non–polar or low-polar compounds can contribute to the activity on TRPA1; this data is in agreement with the previously reported identification of analgesic effects also in non-polar extracts [24].

The concentration of moringin in *Moringa oleifera* preparations is dependent by the presence of endogenous myrosinase and it has been shown to diminish in formulations as powder dry leaves, where the enzyme has been inactivated by thermal treatment. For food supplements, the use of fresh leaves, or extracts from fresh or frozen leaves allows to maximize the concentration of the TRPA1 active compound moringin. As an alternative, the in situ formation of moringin from glucomoringin could be promoted by chemical hydrolysis and this could be a suggestion for further research directions and applications.

## 4. Materials and Methods

### 4.1. Plant Material

Moringa leaves were collected in 2018 from trees experimentally cultivated at Centro Experimental de Formación Agrícola (CEFA), Ministerio de Desarrollo Económico, República Árabe Saharaui Democrática, an oasis created in 2009 next to Rabouni camp (Tindouf, South-Western Algeria). The leaves were dried through a shade-dried process at room temperature, and ground to a fine powder with an electric grinder. One portion was used for preparation of extracts E1-4. Another portion of fresh leaves was collected and immediately frozen after transportation. This sample of frozen leaves has been used for the preparation of extracts E5 and E6.

### 4.2. Chemicals and Equipment

NMR spectra were recorded on a Bruker Avance spectrometer operating at 600 MHz. Samples were dissolved in deuterated methanol (MetOH-*d*_4_) and analyzed; J values are given in Hertz. Thin-layer chromatography (TLC) was performed on silica gel 60 F254 plates (Sigma-Aldrich, Milano, Italy) and developed using a mixture of ethyl acetate/methanol 98/2 as a mobile phase. The spots were visualized under UV light at 254 nm.

HPLC analysis were performed with a liquid Chromatograph Dynamax SD200 (VARIAN^®^, Woburn, MA, USA), equipped with a binary pump with Rheodyne injector and a UV-VIS detector managed by the Galaxy chemstation (VARIAN, Woburn, MA, USA). A reversed phase column C18 ODS Hypersil (250 mm length, 4.6 mm ID, 5 μ, Phenomenex^®^, Castel Maggiore, Italy) was used. The samples were dissolved in methanol and filtered with 0.45 μm nylon filters. The conditions used are the following: flow 1 mL/min, λ 254 nm; solution A: trifluoroacetic acid 0.1%; solution B: acetonitrile; gradient elution from B 20% to B 60% in 35 min, then B 100% in 45 min. In this condition moringin **2** has a retention time of 18.35 min. FTIR spectra was registered on ATR 4600 Jasco instrument (Jasco Europe, Cremella, Italy). ESI-MS mass determination was performed at the COSPECT centre of the University of Milan, with a mass spectrometer ESI-Q-Tof: SYNAPT G2-Si HDMS 8k, Waters SpA, working with Masslynx software (version 2.0) and coupled with Waters Ultra Performance LC System Acquity UPLC H-Class (Waters, Sesto San Giovanni, Italy).

### 4.3. Preparation of Extracts

An aliquot of 5.03 g of dried powder of moringa leaves was extracted at room temperature, in sequence, with magnetic stirring with four solvents with increasing polarity: hexane, diethyl ether, dichloromethane and methanol. A drug/solvent ratio of 1/10 was used and each extraction was done twice for each solvent and each step was proceeded for 2 and a half hours. At the end of each session, the extract was filtered and collected in a volumetric flask and the solvents evaporated in vacuo. Four crude extract were obtained respectively from hexane (E1: 79.6 mg, 1.59%), diethylether (E2: 15.3 mg, 0.30%), dichloromethane (E3: 62.4 mg, 1.24%) and methanol (E4: 180.2 mg, 3.58%).

For extracts E5 and E6, the extraction of moringa leaves was performed to optimize in situ biotrasformation of glucosinolates into the corresponding isothiocyanates by myrosinase. The extracts were prepared from frozen leaves (10 g) in water and methanol respectively.

Frozen leaves (10 g) were finely crushed and resuspended in 50 mL of Millipore water for 30 min at room temperature. After filtration the solvent was lyophilized to obtain extract E5 (0.6994 g, 6.99%). Frozen leaves (10 g) were finely crushed and resuspended in 20 mL of methanol for 4 h at room temperature. After filtration the solvent was evaporated to obtain extract E6 (0.126 g, 1.26%).

The extractions were conducted according to Waterman [47].

### 4.4. Isolation and Purification of Moringin

Moringin was extracted from ground frozen leaves (10 g) in MeOH (20 mL) at room temperature for 4 h. The methanolic extract was evaporated *in vacuo* and partitioned in water: hexane 1:1 three times with 20 mL of each solvent. Finally, water phase was partitioned with ethyl acetate. Organic phase was dried and the solvent evaporated in vacuo to obtain a crude extract (0.0735 g). Purification was carried out by flash chromatography (ethyl acetate/methanol 98:2). Pure moringin was obtained as a colorless gummy powder (15 mg, 0.15%).

TLC Rf 0.25 (silica gel, ethyl acetate/methanol 98/2 *v*/*v*)

^1^H NMR (MeOH-*d*_4_) δ: 1.22 (3H, d, *J* = 6.1 Hz, CH_3_), 3.46 (1H, t, *J* = 9.5, H-4′), 3.63 (1H, m, H-5′), 3.84 (1H, dd, *J* = 3.5 and 9.5 Hz, H-3′), 4.00 (1H, bs, H-2′), 4.69 (2H, s, CH_2_), 5.44 (1H, bs, H-1′), 7.09 and 7.29 (4H, 2d, J = 8.4 Hz, arom.). ^13^C NMR (MeOH-d4) δ: 13.1, 46.7, 68.9, 70.2, 70.5, 72.0, 98.1, 116.1, 127.8, 128.2, 127.9, 155.9.

FTIR ν_max_ 3381, (br. OH), 2920, 2172, 2086, 1609, 1609, 1508, 1236, 1119, 1123, 1058, 983 cm^−1^.

ESI-MS 334.0723 [M + Na]^+^, 100%, (theoretical C_14_H_17_NO_5_SNa 334.0720); 335.0750 [M + 1 + Na]^+^, 20%; 336.0710 [M + 2 + Na]^+^, 4%. Analytical data of moringin are shown in Supplementary material.

### 4.5. In Vitro Assays with rTRP Ion Channels

Compound and extracts’ effects on intracellular Ca^2+^ concentration ([Ca^2+^]_i_) were determined using the selective intracellular fluorescent probe for Ca^2+^ Fluo-4. Assay of rat TRPA1, TRPV2, TRPV3, TRPV4, TRPM8 or human TRPV1 mediated elevation of intracellular Ca^2+^ in transfected HEK-293 cells are been performed as described [56]. Briefly, human embryonic kidney (HEK-293) cells, stably transfected with rat TRPA1, TRPV2, TRPV3, TRPV4, TRPM8 or human TRPV1 (selected by Geneticin 600 μg mL^−1^) or not transfected, were cultured in EMEM + 2 mM Glutamine + 1% Non-Essential Amino Acids (NEAA) + 10% FBS and maintained at 37 °C with 5% CO_2_. Stable transfections were checked by quantitative real-time polymerase chain reaction (PCR), quantitative real-time PCR was performed by an iCycler-iQ (Bio-Rad, Hercules, CA, USA) in a 20 μL reaction mixture containing the following: 10 μL iQ SYBR Green Supermix 2X (Bio-Rad), 20 ng of cDNA (calculated on the basis of the retro-transcribed RNA), and 330 nM for each primer. Optimized primers for SYBR Green analysis (and relative TaOpt) and optimum annealing temperatures were designed by Allele-Id software version 7.0 (Biosoft International, Palo Alto, CA, USA) and were synthesized (HPLC-purification grade) by MWG-Biotech AG (Ebersberg, Germany). Assays were performed in quadruplicate (maximal ΔCt of replicate samples, <0.5). Relative expression analysis, correct for PCR efficiency and normalized with respect to reference genes β-actin and glyceraldehyde 3-phosphate dehydrogenase, was performed by GENEX software (Bio-Rad). TRP-HEK-293 cells express high levels of TRP transcripts, while these transcripts were virtually absent in wild type HEK-293 cells. The day of the experiment the cells were loaded with Fluo-4 AM (4 μM in DMSO containing 0.02% Pluronic F-127) for 1 h in the dark at room temperature, rinsed and resuspended in Tyrode’s solution (145 mM NaCl, 2.5 mM KCl, 1.5 mM CaCl_2_, 1.2 mM MgCl_2_, 10 mM d-glucose, and 10 mM HEPES, pH 7.4), finally transferred to a quartz cuvette of a spectrofluorimeter (Perkin-Elmer LS50B; λEX = 488 nm, λEM = 516 nm) equipped with PTP-1 Fluorescence Peltier System (PerkinElmer Life and Analytical Sciences, Waltham, MA, USA) under continuous stirring. Cell fluorescence before and after the addition of various concentrations of test compounds and extracts were measured normalizing the effects against the response to ionomycin (4 µM). The values of the effect on [Ca^2+^]_i_ in HEK-293 cells not transfected are used as baseline and subtracted from the values obtained from transfected cells. For TRPA1 agonist efficacy was expressed as a percentage of the effect on [Ca^2+^]_i_ observed with 100 μM allylisothiocyanate (AITC). In our experiment on rTRPA1 HEK-293 cells 100 μM allyl isothiocyanate (AITC) exerts an efficacy of 52.4 ± 4.4% of the response to ionomycin 4 µM, while it shows only a negligible effect on not transfected HEK-293 cells (about 1%). The potency of the compounds (EC_50_ values) are determined as the concentration required to produce half-maximal increases in [Ca^2+^]_i_. In TRPV3 assay TRPV3-expressing HEK293 cells were first sensitized with the structurally unrelated agonist 2-aminoethoxydiphenyl borate (100 μM). Antagonist/desensitizing behaviour is evaluated against the agonist of the TRP analyzed by adding the compounds directly in the quartz cuvette 5 min before stimulation of cells with the agonist. In particular, we used AITC 100 μM for TRPA1, capsaicin 0.1μM for TRPV1, lysophosphatidylcholine (LPC) 3μM for TRPV2, thymol 100μM for TRPV3, GSK1016790A 10nM for TRPV4 and icilin 0.25 μM for TRPM8. In order to assess functional antagonism of TRPA1 channels, AP18, 4-(4-chlorophenyl)-3-methyl-3-buten-2-one oxime [57] 50 μM was pre-incubated with the cells, for 5 min before the addition of AITC (100 μM) and moringin (10 μM). IC_50_ is expressed as the concentration exerting a half-maximal inhibition of agonist effect, taking as 100% the effect on [Ca^2+^]_i_ exerted by the agonist alone. Dose-response curve fitting (sigmoidal dose-response variable slope) and parameter estimation were performed with Graph-Pad Prism8^®^ (GraphPad Software Inc., San Diego, CA, USA). All determinations were performed at least in triplicate. Time-course of calcium bound Fluo-4 fluorescence intensity in rTRPA1 experiments are shown in Supplementary material.

## Figures and Tables

**Table 1:** In vitro assays on transient receptor potential ankyrin 1 (TRPA1) ion channel.

	TRPA1		
Sample	Efficacy (% AITC 100 µM)	Potency EC_50_ µg/mL	Inhibition IC_50_ μg/mL (AITC 100 µM)
E1 (hexane)	112.6 ± 4.9	9.0 ± 0.9	25.7 ± 1.4
E2 (ethylether)	104.1 ± 7.7	15.0 ± 2.9	40.9 ± 1.4
E3 (dichloromethane)	64.8 ± 1.7	> 20	> 50
E4 (ethanol)	19.6 ± 1.9	> 20	> 50
E5 (water) ^1^	37.5 ± 1.5	> 20	> 50
E6 (methanol) ^1^	99.0 ± 1.7	1.3 ± 0.1	1.2 ± 0.2
Moringin	102.6 ± 1.1	3.14 ± 0.16 μM	3.60 ± 0.05 μM

^1^ Samples E1-4 from dry leaves; E5 and E6 from frozen leaves. AITC = allylisothiocyanate.

**Table 2:** In vitro assays of moringin on TRPV and TRPM8 ion channels.

	Efficacy (% Ionomycin 4 µM)	Potency ^1^ EC_50_ μM	IC_50_ μM	Reference Agonist
TRPV1	46.8 ± 0.7	20.4 ± 1.3	>50	capsaicin 0.1 μM
TRPV2	<10	n.a.	>100	LPC 3 μM
TRPV3	<10	n.a.	>100	thymol 100 μM
TRPV4	<10	n.a.	>100	GSK1016790A 10 nM
TRPM8	<10	n.a.	>100	icilin 0.25 μM

^1^ When the efficacy is lower than 10% the compound is considered not active (n.a.) and potency cannot be calculated.

## Supplementary Materials

The following are available online at, S1: Analytical data of moringin; S2: Time-course of calcium bound Fluo-4 fluorescence intensity in rTRPA1 experiments.
