# Supplementary material for: Moringin, A Stable Isothiocyanate from Moringa oleifera, Activates the Somatosensory and Pain Receptor TRPA1 Channel In Vitro
Source: Molecules. 2020 Feb 22;25(4):976. doi: 10.3390/molecules25040976 (PMC7070407; doi:10.3390/molecules25040976)
Supplement: Supplementary file 1 [file molecules-25-00976-s001.pdf]

Moringin, a stable isothiocyanate from Moringa oleifera, activates the somatosensory and pain receptor TRPA1 channel in vitro, by *Gigliola Borgonovo, Luciano De Petrocellis, Aniello Schiano Moriello, Simona Bertoli, Alessandro Leone, Alberto Battezzati, Stefania Mazzini, Angela Bassoli.*

S1- supplementary material- Analytical data for isolated moringin and in vitro assays.

1H-NMR

1H NMR (MeOH-d4) δ: 1.22 (3H, d, J= 6.1 Hz, CH3), 3.46 (1H, t, J= 9.5, H-4'), 3.63 (1H, m, H-5'), 3.84 (1H, dd, J= 3.5 and 9.5 Hz, H-3'), 4.00 (1H, bs, H-2'), 4.69 (2H, s, CH2), 5.44 (1H, bs, H-1'), 7.09 and 7.29 (4H, 2d, J= 8.4 Hz, arom.).

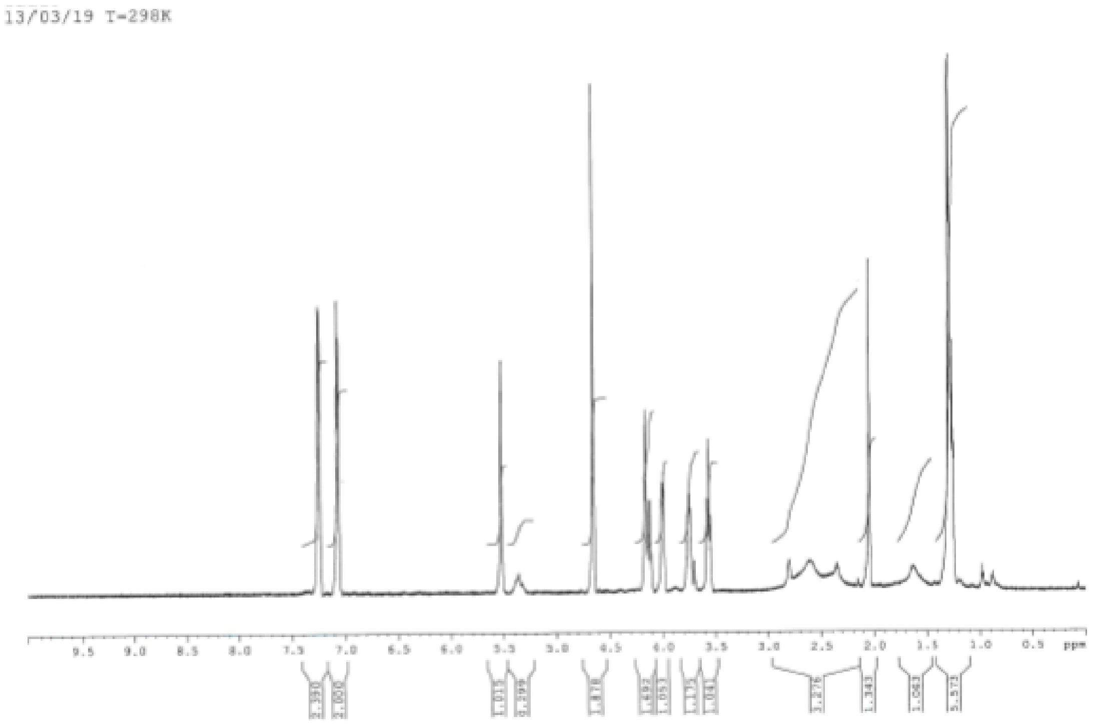

13C-NMR spectrum

13C NMR (MeOH-d4) δ: 13.1, 46.7, 68.9, 70.2, 70.5, 72.0, 98.1, 116.1, 127.8, 128.2, 127.9, 155.9.

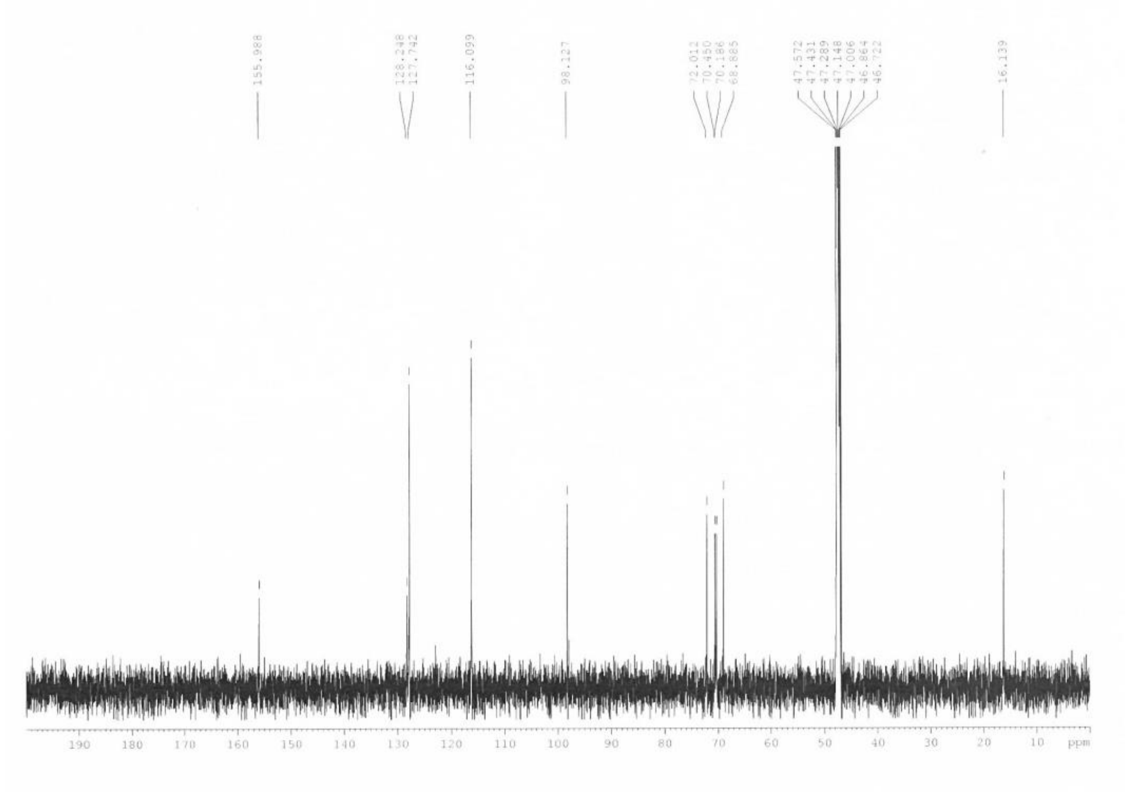

Mass spectrum, TOF MS ES

ESI-MS 334.0723 [M + Na]+ , 100%, (theoretical C14H17NO5SNa 334.0720); 335.0750 [M +1+Na]+, 20%; 336.0710 [M +2+Na]+, 4%.

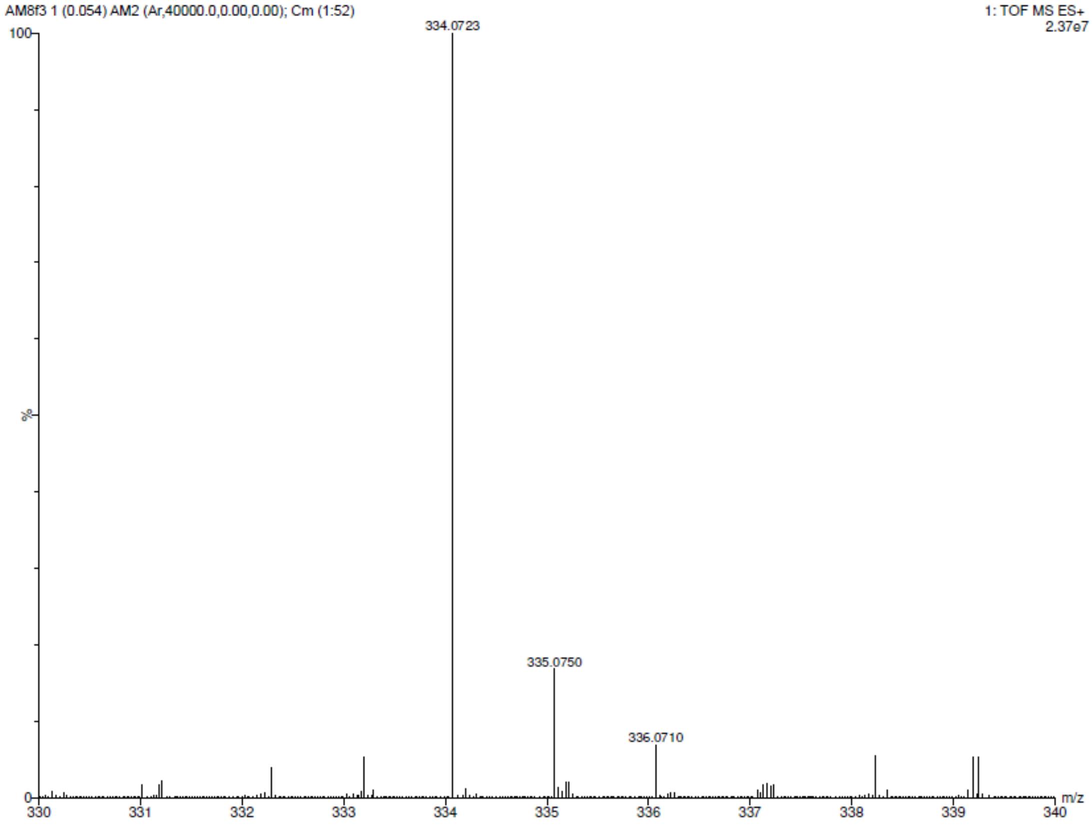

FTIR spectrum

FTIR vmax 3381, (br. OH), 2920, 2172, 2086, 1609, 1609, 1508, 1236, 1119, 1123, 1058, 983 cm-1.

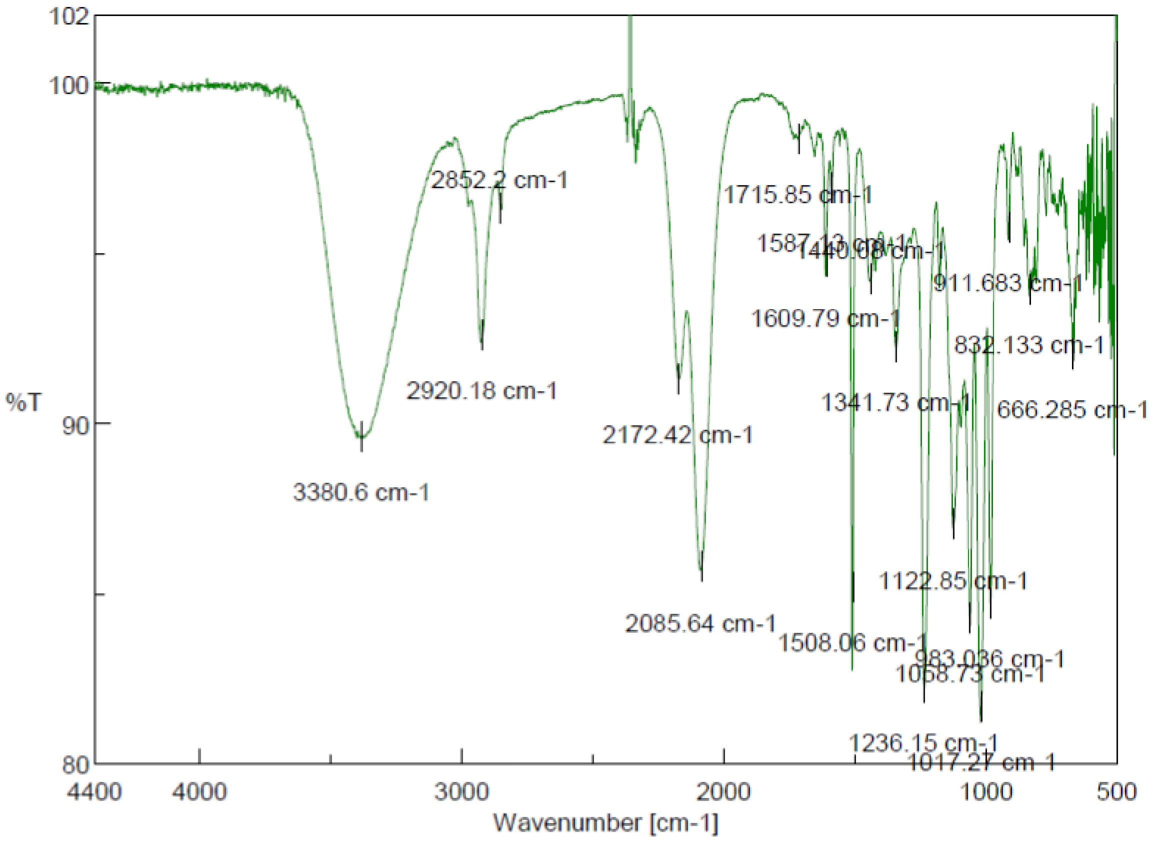

Time-course of calcium bound Fluo-4 fluorescence intensity in rTRPA1 experiments

In our experiment on rTRPA1 HEK-293 cells 100 µM allyl isothiocyanate (AITC) exerts an efficacy of 52.4 ± 4.4 % of the response to ionomycin 4 µM. Moringin in not transfected HEK-293 cells exert only a very little increase of $[Ca^{2+}]_i$ (<< 10 % of the response in rTRPA1 HEK-293 cells) and this effect was used as baseline and subtracted from the values obtained from transfected cells.

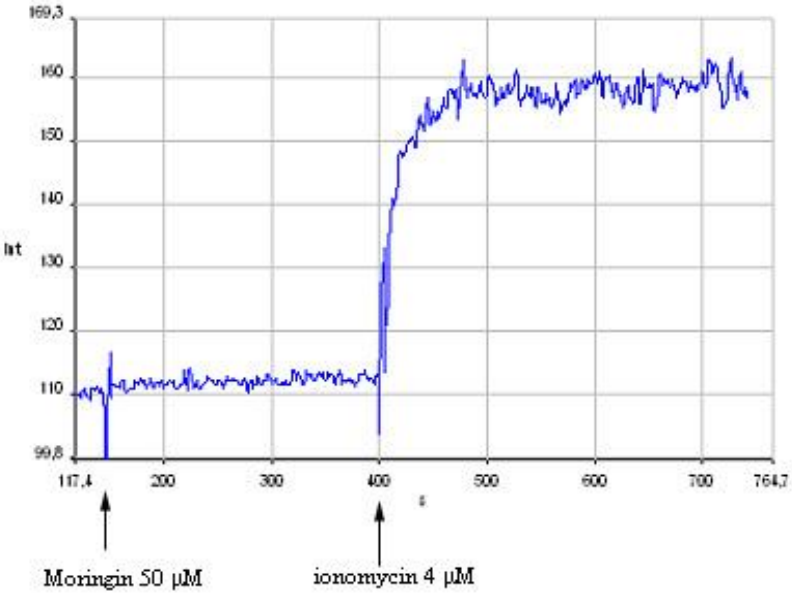

HEK-293 wt

The effect of AITC 100 µM administered after a pre-incubation of TRPA1-HEK293 cells with moringin is completely inhibited.

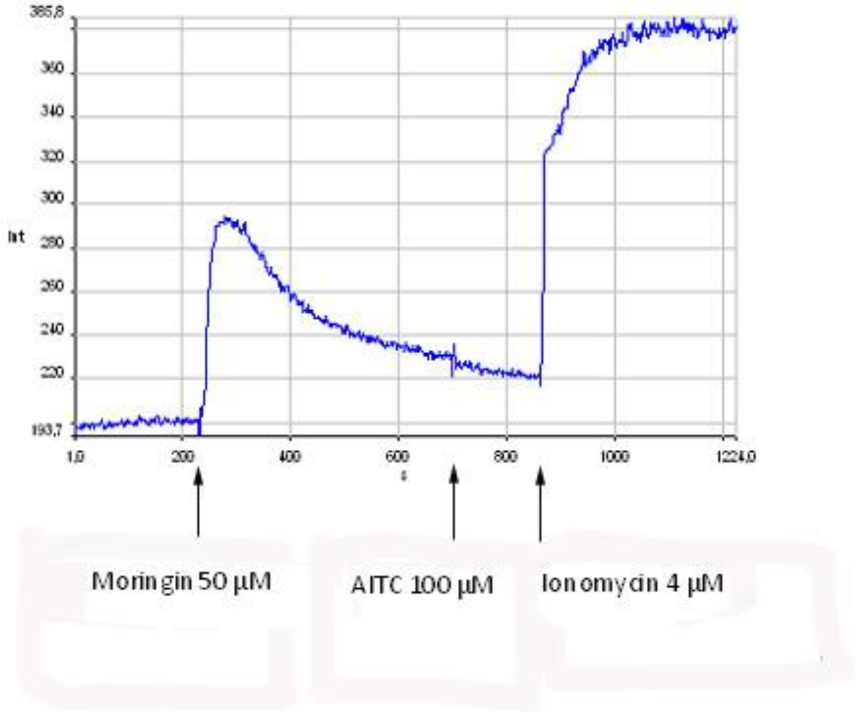

TRPA1-HEK-293
